# Sugar consumption and early childhood caries: a systematic review and meta-analysis of cohort studies

**DOI:** 10.1590/1807-3107bor-2025.vol39.122

**Published:** 2025-11-17

**Authors:** Mariana Silveira ECHEVERRIA, Fernanda Burkert MATHIAS, Helena Silveira SCHUCH, Maximiliano Sérgio CENCI, Marcos Britto CORREA, Marie-Charlotte HUYSMANS, Flávio Fernando DEMARCO

**Affiliations:** (a) Universidade Federal de Pelotas – UFPel, Graduate Program in Epidemiology, Pelotas, RS, Brazil.; (b) Radboud University Medical Center, Department of Dentistry, Nijmegen, The Netherlands.

**Keywords:** Sugars, Dental Caries, Epidemiology, Systematic Review, Meta-analysis

## Abstract

The aim of this systematic literature review and meta-analysis was to answer the following research question: “Is sugar consumption associated with early childhood caries among children under 6 years of age in cohort studies?”. The following electronic databases were accessed from December 2020 to May 2025 to identify the existing literature: Bireme, Pubmed/Medline, Scielo, Scopus, and Web of Science. Studies considered eligible for this systematic review were those that investigated sugar consumption as the main exposure and early childhood caries (ECC) as the outcome. Study selection and data extraction were performed independently by two reviewers. Study quality was assessed using the Joanna Briggs Institute (JBI) checklist for cohort studies scale. The search strategy retrieved 718 studies. After title and abstract screening, 59 were selected for full-text review, leading to the inclusion of 17 original studies in this systematic review. Finally, nine studies provided sufficient data for a meta-analysis. The association between sugar consumption and ECC was consistent across most of the cohort studies included in this review, which reported that higher sugar consumption was associated with higher prevalence of dental caries. The pooled effect estimate from the meta-analysis yielded an OR of 1.59 and a 95% CI (1.50-1.68), indicating that children who consumed sugar in early childhood were 59% more likely to develop caries compared to those who did not consume sugar. The included studies had a low risk of bias. Our systematic review and meta-analysis confirmed the association between sugar consumption and ECC in longitudinal cohort studies.

## Introduction

Dental caries is the most common chronic disease in childhood, affecting around 621 million children worldwide,^1^ with a prevalence greater than 50% among those under 6 years of age in most countries.^1,2^ Given this alarming prevalence, in addition to the impact of dental caries on children’s health and well-being,^3^ it is necessary to identify the determinants of early childhood caries (ECC) to guide preventive interventions,^4^ which are the most promising strategies for addressing this public health issue. Longitudinal studies have evidenced that sugar intake during the first few years of life is associated with ECC.^5-8^ Therefore, preventive interventions should focus on reducing the widely acknowledged primary cause of dental caries: presence of added sugars in the diet.^9^

The recommendation from the World Health Organization (WHO) is to limit the consumption of free sugars by up to 5% of the total energy intake to reduce obesity, caries, and other chronic non-communicable diseases in children and adults.^10^ In the Bangkok Declaration, a document from the International Association of Pediatric Dentistry (IAPD), the recommendation is to limit the consumption of sugar in food and beverages and avoid free sugars for children under 2 years of age to reduce the prevalence and impact of ECC.^11^

Sugar continues to be highly consumed from the first year of life.^5-8^ Given that dental caries is linked to sugar intake, the literature consistently shows that a diet excessively high in sugar, particularly sucrose, is the main cause of dental caries.^12^ Some evidence supports the relationship between sugar and dental caries; however, such relationship is still controversial. For instance, different terms are used in the scientific literature to describe sugar intake.^9^ In addition, the association between sugar intake and dental caries was evaluated in different populations and age groups using different designs and methodological approaches. Longitudinal studies, such as cohort studies, are the most appropriate approach to assess the impact of an exposure during the life course on a given outcome,^13^ but investigation into the association between sugar and dental caries using longitudinal data is still scarce, especially considering data from low- and medium-income countries, where dental caries is more prevalent.

Accordingly, this systemic review and meta-analysis aimed to investigate the association between sugar consumption and ECC in primary dentition using data from longitudinal studies.

## Methods

A systematic literature review was performed to identify existing cohort studies on the effect of sugar consumption on dental caries. The review aimed to answer the following research question: “Is sugar consumption associated with early childhood caries among children under 6 years of age in cohort studies?”. The research question was constructed according to the PICO strategy: Participants/population: children under 6 years of age; Intervention/exposure: sugar intake; Control/comparator: no sugar consumption; and Outcome: early childhood caries. This study was reported following the PRISMA (Preferred Reporting Items for Systematic Reviews and Meta-Analyses) guidelines for systematic reviews.^14^ The review protocol was registered on the International prospective register of systematic reviews (PROSPERO) platform under number CRD42021232374.

Five electronic databases were accessed to identify the existing literature on the subject: Bireme, Pubmed/Medline, Scielo, Scopus, and Web of Science. A search strategy was built based on the research question and adapted for each database, as shown in Table 1. Searches were merged using Rayyan (https://www.rayyan.ai), and duplicates were removed. The review was carried out independently by two reviewers (MSE and FBM) from December 2020 to May 2025 by applying the inclusion and exclusion criteria. Cohort studies were considered eligible for this systematic review if sugar consumption was the main exposure and ECC was the outcome. The following exclusion criteria were applied: a) studies not aligned with the established PICO and those in which sugar was treated only as a covariate; b) non-cohort studies; c) studies including participants from age groups outside the scope of the present study, i.e., samples involving individuals aged 6 years or older; d) studies performed using convenience samples; and e) studies that analyzed dental caries based on parental reports only, without clinical evaluation. Titles and abstracts were initially screened, and eligible studies were then read in full. In case of discrepancies in the assessments, the reviewers discussed them until they could reach a consensus. Persistent disagreements were discussed with a third reviewer (HSS). Interrater agreement was calculated using Cohen’s kappa coefficient, which yielded a value of 0.81.

**Table 1:** Systematized database search strategies for studies on sugar consumption and early childhood caries.

Database	Search key	Number of articles found
Pubmed	((((((((((((((((((((((((((((((((((((((((Early Childhood Caries [all fields]) OR (Early Childhood Caries [MeSH Terms])) OR (dental caries [MeSH Terms])) OR (dental caries [All Fields])) OR (dental decay [MeSH Terms])) OR (dental decay [all fields])) OR (Decay, Dental [all fields])) OR (Decay, Dental [MeSH Terms])) OR (Carious Lesions [MeSH Terms])) OR (Carious Lesions[All Fields])) OR (Carious Lesion[MeSH Terms])) OR (Carious Lesion[All Fields])) OR (Lesion, Carious[MeSH Terms])) OR (Lesion, Carious[All Fields])) OR (Lesions, Carious[MeSH Terms])) OR (Lesions, Carious[All Fields])) OR (Caries, Dental[MeSH Terms])) OR (Caries, Dental[All Fields])) OR (Carious Dentin[MeSH Terms])) OR (Carious Dentin[All Fields])) OR (Carious Dentins[MeSH Terms])) OR (Carious Dentins[All Fields])) OR (Dentin, Carious[MeSH Terms])) OR (Dentin, Carious[All Fields])) OR (Dentins, Carious[MeSH Terms])) OR (Dentins, Carious[All Fields])) OR (Dental White Spot[MeSH Terms])) OR (Dental White Spot[All Fields])) OR (Spot, Dental White[MeSH Terms])) OR (Spot, Dental White[All Fields])) OR (Spots, Dental White[MeSH Terms])) OR (Spots, Dental White[All Fields])) OR (White Spot, Dental[MeSH Terms])) OR (White Spot, Dental[All Fields])) OR (White Spots, Dental[MeSH Terms])) OR (White Spots, Dental[All Fields])) OR (Dental White Spots[MeSH Terms])) OR (Dental White Spots[All Fields])) AND ((((((((((((((((((((((((((((((((((((((((((((((((((((((child*[MeSH Terms]) OR (child*[all fields])) OR (Preschool Child[MeSH Terms])) OR (Preschool Child[all fields])) OR (Children, Preschool[MeSH Terms])) OR (Children, Preschool[all fields])) OR (Preschool Children[MeSH Terms])) OR (Preschool Children[all fields])) OR (deciduous tooth[MeSH Terms])) OR (deciduous tooth[all fields])) OR (Dentition, Deciduous[MeSH Terms])) OR (Dentition, Deciduous[all fields])) OR (Deciduous Dentition[MeSH Terms])) OR (Deciduous Dentition[all fields])) OR (Deciduous Dentitions[MeSH Terms])) OR (Deciduous Dentitions[all fields])) OR (Dentitions, Deciduous[MeSH Terms])) OR (Dentitions, Deciduous[All Fields])) OR (Dentition, Primary[MeSH Terms])) OR (Dentition, Primary[All Fields])) OR (Dentitions, Primary[MeSH Terms])) OR (Dentitions, Primary[All Fields])) OR (Primary Dentition[MeSH Terms])) OR (Primary Dentition[All Fields])) OR (Primary Dentitions[MeSH Terms])) OR (Primary Dentitions[All Fields])) OR (Milk Tooth[MeSH Terms])) OR (Milk Tooth[All Fields])) OR (Tooth, Milk[MeSH Terms])) OR (Tooth, Milk[All Fields])) OR (Primary Teeth[MeSH Terms])) OR (Primary Teeth[All Fields])) OR (Teeth, Deciduous[MeSH Terms])) OR (Teeth, Deciduous[All Fields])) OR (Deciduous Teeth[MeSH Terms])) OR (Deciduous Teeth[All Fields])) OR (Teeth, Primary[MeSH Terms])) OR (Teeth, Primary[All Fields])) OR (Tooth, Primary[MeSH Terms])) OR (Tooth, Primary[All Fields])) OR (Milk Teeth[MeSH Terms])) OR (Milk Teeth[All Fields])) OR (Teeth, Milk[MeSH Terms])) OR (Teeth, Milk[All Fields])) OR (Baby Teeth[MeSH Terms])) OR (Baby Teeth[All Fields])) OR (Teeth, Baby[MeSH Terms])) OR (Teeth, Baby[All Fields])) OR (Baby Tooth[All Fields])) OR (Baby Tooth[MeSH Terms])) OR (Tooth, Baby[MeSH Terms])) OR (Tooth, Baby[All Fields])) OR (Primary Tooth[MeSH Terms])) OR (Primary Tooth[All Fields]))) AND ((((((((((((diet[MeSH Terms]) OR (diets[MeSH Terms])) OR (diet[All Fields])) OR (diets[All Fields])) OR (sugar[MeSH Terms])) OR (sugar[All Fields])) OR (Candy[MeSH Terms])) OR (Candy[All Fields])) OR (Candies[MeSH Terms])) OR (Candies[All Fields])) OR (sugar consumption[MeSH Terms])) OR (sugar consumption[All Fields]))) AND ((((((((((((((((((((((((((((((((((((((((longitudinal studies[MeSH Terms]) OR (longitudinal studies[All Fields])) OR (cohort studies[MeSH Terms])) OR (longitudinal studies[all fields])) OR (cohort studies[ all fields])) OR (Longitudinal Study[all fields])) OR (Studies, Longitudinal[all fields])) OR (Study, Longitudinal[all fields])) OR (Longitudinal Survey[all fields])) OR (Longitudinal Surveys[all fields])) OR (Survey, Longitudinal[all fields])) OR (Surveys, Longitudinal[all fields])) OR (Cohort Study[all fields])) OR (Studies, Cohort[all fields])) OR (Study, Cohort[all fields])) OR (Concurrent Studies[all fields])) OR (Studies, Concurrent[all fields])) OR (Concurrent Study[all fields])) OR (Study, Concurrent[all fields])) OR (Closed Cohort Studies[all fields])) OR (Cohort Studies, Closed[all fields])) OR (Closed Cohort Study[all fields])) OR (Cohort Study, Closed[all fields])) OR (Study, Closed Cohort[all fields])) OR (Studies, Closed Cohort[all fields])) OR (Analysis, Cohort[all fields])) OR (Cohort Analysis[all fields])) OR (Analyses, Cohort[all fields])) OR (Cohort Analyses[all fields])) OR (Historical Cohort Studies[all fields])) OR (Cohort Study, Historical[all fields])) OR (Historical Cohort Study[all fields])) OR (Study, Historical Cohort[all fields])) OR (Study, Historical Cohort[all fields])) OR (Cohort Studies, Historical[all fields])) OR (Studies, Historical Cohort[all fields])) OR (Incidence Studies[all fields])) OR (Incidence Study[all fields])) OR (Studies, Incidence[all fields])) OR (Study, Incidence[all fields]))	623
Scopus	TITLE-ABS-KEY ( «Early Dental Caries» OR «dental caries» OR «dental decay» OR «Decay, Dental» OR «Carious Lesions» OR «Carious Lesion» OR «Lesion, Carious» OR «Lesions, Carious» OR «Caries, Dental» OR «Carious Dentin» OR «Carious Dentins» OR «Dentin, Carious» OR «Dentins, Carious» OR «Dental White Spot» OR «Spot, Dental White» OR «Spots, Dental White» OR «White Spot, Dental» OR «White Spots, Dental» OR «Dental White Spots» ) AND TITLE-ABS-KEY ( «child» OR «Preschool Child» OR «Children, Preschool» OR «Preschool Children» OR «deciduous tooth» OR «Dentition, Deciduous» OR «Deciduous Dentition» OR «Deciduous Dentitions» OR «Dentitions, Deciduous» OR «Dentition, Primary» OR «Dentitions, Primary» OR «Primary Dentition» OR «Primary Dentitions» OR «Milk Tooth» OR «Tooth, Milk» OR «Primary Teeth» OR «Teeth, Deciduous» OR «Deciduous Teeth» OR «Teeth, Primary» OR «Tooth, Primary» OR «Milk Teeth» OR «Teeth, Milk» OR «Baby Teeth» OR «Teeth, Baby» OR «Baby Tooth» OR «Tooth, Baby» OR «Primary Tooth» ) AND TITLE-ABS-KEY ( «diet» OR «diets» OR «sugar» OR «Candy» OR «Candies» OR «sugar consumption» ) AND TITLE-ABS-KEY ( «longitudinal studies» OR «cohort studies» OR «Longitudinal Study» OR «Studies, Longitudinal» OR «Study, Longitudinal» OR «Longitudinal Survey» OR «Longitudinal Surveys» OR «Survey, Longitudinal» OR «Surveys, Longitudinal» OR «Cohort Study» OR «Studies, Cohort» OR «Study, Cohort» OR «Concurrent Studies» OR «Studies, Concurrent» OR «Concurrent Study» OR «Study, Concurrent» OR «Closed Cohort Studies» OR «Cohort Studies, Closed» OR «Closed Cohort Study» OR «Cohort Study, Closed» OR «Study, Closed Cohort» OR «Studies, Closed Cohort» OR «Analysis, Cohort» OR «Cohort Analysis» OR «Analyses, Cohort» OR «Cohort Analyses» OR «Historical Cohort Studies» OR «Cohort Study, Historical» OR «Historical Cohort Study» OR «Study, Historical Cohort» OR «Study, Historical Cohort» OR «Cohort Studies, Historical» OR «Studies, Historical Cohort» OR «Incidence Studies» OR «Incidence Study» OR «Studies, Incidence» OR «Study, Incidence» )	226
Web of Science	ALL=( «Early Dental Caries» OR «dental caries» OR «dental decay» OR «Decay, Dental» OR «Carious Lesions» OR «Carious Lesion» OR «Lesion, Carious» OR «Lesions, Carious» OR «Caries, Dental» OR «Carious Dentin» OR «Carious Dentins» OR «Dentin, Carious» OR «Dentins, Carious» OR «Dental White Spot» OR «Spot, Dental White» OR «Spots, Dental White» OR «White Spot, Dental» OR «White Spots, Dental» OR «Dental White Spots» ) AND ALL=( «child» OR «Preschool Child» OR «Children, Preschool» OR «Preschool Children» OR «deciduous tooth» OR «Dentition, Deciduous» OR «Deciduous Dentition» OR «Deciduous Dentitions» OR «Dentitions, Deciduous» OR «Dentition, Primary» OR «Dentitions, Primary» OR «Primary Dentition» OR «Primary Dentitions» OR «Milk Tooth» OR «Tooth, Milk» OR «Primary Teeth» OR «Teeth, Deciduous» OR «Deciduous Teeth» OR «Teeth, Primary» OR «Tooth, Primary» OR «Milk Teeth» OR «Teeth, Milk» OR «Baby Teeth» OR «Teeth, Baby» OR «Baby Tooth» OR «Tooth, Baby» OR «Primary Tooth» ) AND ALL=( «diet» OR «diets» OR «sugar» OR «Candy» OR «Candies» OR «sugar consumption» ) AND ALL=( «longitudinal studies» OR «cohort studies» OR «Longitudinal Study» OR «Studies, Longitudinal» OR «Study, Longitudinal» OR «Longitudinal Survey» OR «Longitudinal Surveys» OR «Survey, Longitudinal» OR «Surveys, Longitudinal» OR «Cohort Study» OR «Studies, Cohort» OR «Study, Cohort» OR «Concurrent Studies» OR «Studies, Concurrent» OR «Concurrent Study» OR «Study, Concurrent» OR «Closed Cohort Studies» OR «Cohort Studies, Closed» OR «Closed Cohort Study» OR «Cohort Study, Closed» OR «Study, Closed Cohort» OR «Studies, Closed Cohort» OR «Analysis, Cohort» OR «Cohort Analysis» OR «Analyses, Cohort» OR «Cohort Analyses» OR «Historical Cohort Studies» OR «Cohort Study, Historical» OR «Historical Cohort Study» OR «Study, Historical Cohort» OR «Study, Historical Cohort» OR «Cohort Studies, Historical» OR «Studies, Historical Cohort» OR «Incidence Studies» OR «Incidence Study» OR «Studies, Incidence» OR «Study, Incidence» )	51
Bireme	((«Early Dental Caries» OR «Cárie na primeira infância» OR «Caries de la primera infancia» OR «dental caries» OR «cáries dentárias» OR «caries dental» OR «dental decay» OR «Decay, Dental» OR «Carious Lesions» OR «Lesões Cariosas» OR «Lesiones cariosas» OR «Carious Lesion» OR «Lesão Cariosa» OR «Lesión cariosa» OR «Lesion, Carious» OR «Lesions, Carious» OR «Caries, Dental» OR «Cárie dentária» OR «Caries Dental» OR «Carious Dentin» OR «Carious Dentins» OR «Dentin, Carious» OR «Dentins, Carious» OR «Dental White Spot» OR «Mancha Branca Dentária» OR «Mancha blanca dental» OR «Spot, Dental White» OR «Spots, Dental White» OR «White Spot, Dental» OR «White Spots, Dental» OR «Dental White Spots») AND («child» OR «criança» OR «niño» OR «Preschool Child» OR «Criança pré-escolar» OR «Children, Preschool» OR «Preschool Children» OR «deciduous tooth» OR «dente decíduo» OR «diente deciduo» OR «Dentition, Deciduous» OR «Deciduous Dentition» OR «Dentição decídua» OR «Dentición decidua» OR «Deciduous Dentitions» OR «Dentitions, Deciduous» OR «Dentition, Primary» OR «Dentitions, Primary» OR «Primary Dentition» OR «Dentição primária» OR «Dentición primaria» OR «Primary Dentitions» OR «Milk Tooth» OR «Dente de leite» OR «Diente de leche» OR «Tooth, Milk» OR «Primary Teeth» OR «Teeth, Deciduous» OR «Deciduous Teeth» OR «Teeth, Primary» OR «Tooth, Primary» OR «Milk Teeth» OR «Teeth, Milk» OR «Baby Teeth» OR «Teeth, Baby» OR «Baby Tooth» OR «Tooth, Baby» OR «Primary Tooth») AND («diet» OR «dieta» OR «diets» OR «sugar» OR «açúcar» OR «azúcar» OR «Candy» OR «Doce» OR «Dulce» OR «Candies» OR «sugar consumption» OR «consumo de açúcar» OR «consumo de azucar») AND («longitudinal studies» OR «estudos longitudinais» OR «estudios longitudinales» OR «cohort studies» OR «estudos de coorte» OR «Longitudinal Study» OR «Estudo longitudinal» OR «Estudio longitudinal» OR «Studies, Longitudinal» OR «Study, Longitudinal» OR «Longitudinal Survey» OR «Longitudinal Surveys» OR «Survey, Longitudinal» OR «Surveys, Longitudinal» OR «Cohort Study» OR «Studies, Cohort» OR «Study, Cohort» OR «Concurrent Studies» OR «Studies, Concurrent» OR «Concurrent Study» OR «Study, Concurrent» OR «Closed Cohort Studies» OR «Cohort Studies, Closed» OR «Closed Cohort Study» OR «Cohort Study, Closed» OR «Study, Closed Cohort» OR «Studies, Closed Cohort» OR «Analysis, Cohort» OR «Cohort Analysis» OR «Analyses, Cohort» OR «Cohort Analyses» OR «Historical Cohort Studies» OR «Cohort Study, Historical» OR «Historical Cohort Study» OR «Study, Historical Cohort» OR «Study, Historical Cohort» OR «Cohort Studies, Historical» OR «Studies, Historical Cohort» OR «Incidence Studies» OR «Incidence Study» OR «Studies, Incidence» OR «Study, Incidence»))	259
Scielo	((«Early Dental Caries» OR «Cárie na primeira infância» OR «Caries de la primera infancia» OR «dental caries» OR «cáries dentárias» OR «caries dental» OR «dental decay» OR «Decay, Dental» OR «Carious Lesions» OR «Lesões Cariosas» OR «Lesiones cariosas» OR «Carious Lesion» OR «Lesão Cariosa» OR «Lesión cariosa» OR «Lesion, Carious» OR «Lesions, Carious» OR «Caries, Dental» OR «Cárie dentária» OR «Caries Dental» OR «Carious Dentin» OR «Carious Dentins» OR «Dentin, Carious» OR «Dentins, Carious» OR «Dental White Spot» OR «Mancha Branca Dentária» OR «Mancha blanca dental» OR «Spot, Dental White» OR «Spots, Dental White» OR «White Spot, Dental» OR «White Spots, Dental» OR «Dental White Spots») AND («child» OR «criança» OR «niño» OR «Preschool Child» OR «Criança pré-escolar» OR «Children, Preschool» OR «Preschool Children» OR «deciduous tooth» OR «dente decíduo» OR «diente deciduo» OR «Dentition, Deciduous» OR «Deciduous Dentition» OR «Dentição decídua» OR «Dentición decidua» OR «Deciduous Dentitions» OR «Dentitions, Deciduous» OR «Dentition, Primary» OR «Dentitions, Primary» OR «Primary Dentition» OR «Dentição primária» OR «Dentición primaria» OR «Primary Dentitions» OR «Milk Tooth» OR «Dente de leite» OR «Diente de leche» OR «Tooth, Milk» OR «Primary Teeth» OR «Teeth, Deciduous» OR «Deciduous Teeth» OR «Teeth, Primary» OR «Tooth, Primary» OR «Milk Teeth» OR «Teeth, Milk» OR «Baby Teeth» OR «Teeth, Baby» OR «Baby Tooth» OR «Tooth, Baby» OR «Primary Tooth») AND («diet» OR «dieta» OR «diets» OR «sugar» OR «açúcar» OR «azúcar» OR «Candy» OR «Doce» OR «Dulce» OR «Candies» OR «sugar consumption» OR «consumo de açúcar» OR «consumo de azucar») AND («longitudinal studies» OR «estudos longitudinais» OR «estudios longitudinales» OR «cohort studies» OR «estudos de coorte» OR «Longitudinal Study» OR «Estudo longitudinal» OR «Estudio longitudinal» OR «Studies, Longitudinal» OR «Study, Longitudinal» OR «Longitudinal Survey» OR «Longitudinal Surveys» OR «Survey, Longitudinal» OR «Surveys, Longitudinal» OR «Cohort Study» OR «Studies, Cohort» OR «Study, Cohort» OR «Concurrent Studies» OR «Studies, Concurrent» OR «Concurrent Study» OR «Study, Concurrent» OR «Closed Cohort Studies» OR «Cohort Studies, Closed» OR «Closed Cohort Study» OR «Cohort Study, Closed» OR «Study, Closed Cohort» OR «Studies, Closed Cohort» OR «Analysis, Cohort» OR «Cohort Analysis» OR «Analyses, Cohort» OR «Cohort Analyses» OR «Historical Cohort Studies» OR «Cohort Study, Historical» OR «Historical Cohort Study» OR «Study, Historical Cohort» OR «Study, Historical Cohort» OR «Cohort Studies, Historical» OR «Studies, Historical Cohort» OR «Incidence Studies» OR «Incidence Study» OR «Studies, Incidence» OR «Study, Incidence»))	3
Total		1162

Study quality was assessed using the Joanna Briggs Institute (JBI) checklist for cohort studies scale. This scale evaluates 11 categories, with yes, no, and unclear as answer options. The overall score of each study was identified by the sum of “yes” responses. Studies with scores from 0 to 5 were classified as high risk of bias, and those with scores ranging from 6 to 10 were rated as low risk of bias.^15^

Information was extracted independently from the manuscripts by the two reviewers and stored in a spreadsheet containing the following information: authors, year of publication, sample size and characteristics, exposure and outcome assessment instruments, variables used to control for confounding factors, main findings, and all items included in the risk of bias assessment.

For the nine studies included in the meta-analysis, effect measures expressed as prevalence ratio (PR) and relative risk (RR) were converted to odds ratio (OR) for standardization of the data. The random-effects model was chosen. Data were analyzed using the Stata 15.0 software (Stata Corp, College Station, TX, USA).

## Results

**Figure 1:**
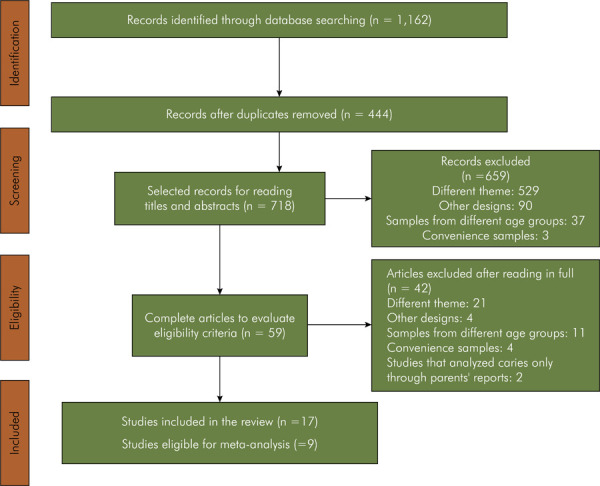
Article selection flowchart.

The search strategy retrieved 1,162 studies. Of these, 444 studies were removed for being duplicates. The titles and abstracts of the remaining 718 articles were read, applying the eligibility criteria outlined in the Methods section. After that, 59 articles were still considered potentially eligible and had their full texts evaluated. Finally, 17 original studies were considered eligible and included in this systematic review. Standard measures were available in nine studies, allowing for their inclusion in the meta-analysis. Figure 1 presents the study selection flowchart, as recommended by the PRISMA guidelines.

The studies included in this systematic review were published from 1985 to 2023. In terms of economic background, most studies were carried out in high-income countries. No studies were conducted in low-income countries, and six were performed in developing countries (Brazil and Thailand). Five studies included in this review were conducted in European countries, five in Brazil, four in Asian countries, three in Australia, and one in the USA.

Sugar exposure before the age of one year was reported in 11 studies.^5-8,16-22^ The study that evaluated sugar consumption in older children assessed it at 36, 48, and 60 months.^23^ Four studies analyzed the patterns of sugar consumption.^5,7,20,24^

The methods used to measure sugar consumption varied across the selected studies. The high frequency of sugars was investigated in eight studies.^16,18,19,23,25-27^ Two studies evaluated the weekly frequency of sugar consumption.^16,25^ Four articles investigated the daily frequency of sugar-containing foods or beverages.^19,23,24,27^ Other studies evaluated sugar intake through food diaries with a 24-hour recall.^17,19,24,25^ In one of the studies, parents/caregivers were instructed to record all food and drink consumption during two weekdays and one weekend day.^23^ Three studies evaluated the introduction of sugary foods in children’s diets.^6,8,22^

Considering the 17 selected studies, the outcomes related to ECC were evaluated from 9 months to 5 years of age, with variations between the analyzed studies. To assess the prevalence of ECC, 11 studies investigated lesions at the initial stage (white spot lesions), while the remaining six studies considered only cavitated lesions. Four articles reported using the International Caries Detection and Assessment System (ICDAS) index as the instrument to measure the outcome,^5,6,16,19^ seven studies evaluated the outcome through the dental caries outcome by the number of decayed, missing, or filled surfaces (dmfs index),^7,8,17,18,20,24,25^ and one study evaluated dental caries using the criteria proposed by Pitts.^23^ The remaining studies (n=5) did not specify how ECC was measured.

Much of the literature reviewed (n = 15) reported an association between sugar intake and ECC, with the strongest effect found for candy consumption (OR 2.28, 95% CI: 1.28–4.04).^25^ This association was not confirmed in two studies included in this review. In one of the studies that did not report an association, it was observed that following a diet based on WHO guidelines was a protective factor for the occurrence of ECC.^19^ In another study, the association between the patterns of consumption of sugary foods and ECC was lost after adjusting for covariates.^20^ The studies included in this review are shown in Table 2. All the cohort studies included had a low risk of bias. The quality assessment of the studies is shown in Table 3.

**Table 2:** Overview of the studies included in the review. n = 17.*

Author, year, country	Sample	Wave	Main exposure (sugar)	Outcome	Confounding factors	Adjusted RR	Main results
Amezdroz et al., 2019, Australia^16^	170 children (VicGen birth cohort study)	6, 12, and 18 months and 5 years of age	Intake levels of foods and beverages characterized as never, low, moderate, high, and very high, determined by weekly frequency of consumption and assigned a value from 0 (never) to 4 (severe)	ICDAS II at 5 years of age: A healthy group who had no ECC as determined by ICDAS scores of 0; a mild-moderate disease group who had one or more lesions with an ICDAS score of 4 or less; and an advanced disease group who had one or more ICDAS scores of 5 or higher.	Not reported	Not reported	The mean cariogenic scale score at the age of 18 months was significantly higher in children aged 5 years in the advanced disease group who had a mean cariogenic scale score of 59.0±15.9 compared to those in the healthy group who had a mean score of 47.7±17.5 or those in the mild-moderate disease group who had a mean score of 48.2±17.3. There was no difference in mean cariogenic scale score between the healthy and mild-moderate disease groups.
			A score for each discretionary food and everage was generated by multiplying the cariogenicity weight (2-moderate, 3-high, or 4-severe) by consumption frequency (0, 1, 2, 3, or 4).				
			The scores for all discretionary food and beverages were summed to produce an overall cariogenicity score between 0 and 276 for each child at 6, 12, and 18 months of age.				
Bernabe et al., 2020, Scotland^7^	1,099, 1,019, 871, and 957 in sweeps 1, 2, 3, and 4, respectively	age 12 to 48 months annually	SSBs intake. SSBs were defined as any liquids containing added caloric sweeteners, such as soft drinks, fruit drinks, energy and sports drinks, and drinks sweetened after purchase (Miller et al. 2013). In every survey, parents reported how many times a day, on average, their children were given sugar-containing hot beverages and sugar-containing cold beverages. The child’s daily intake of SSBs was calculated as the sum of both responses and expressed as times per day. SSB intake was therefore a time- varying predictor (up to four data points per child).	dmfs: including both noncavitated and cavitated lesions assessment from 12 to 48 months	Maternal predictors: age at delivery, smoking during pregnancy, education, parental employment, and level of socioeconomic deprivation of the family’s residential area. Child predictors: sex, age (months), birth weight, breastfeeding, and toothbrushing frequency	Not reported	Data from Scottish young children indicate that the introduction of SSBs during the first year of life may predispose children to high levels of dental caries.
Chaffee et al., 2015, Brazoç^8^	458 chidren, prospective birth cohort in Porto Alegre	6, 12, and 38 months	Food and beverage consumption pattern before 12 months of age	Severe early childhood caries (S-ECC) was defined as ≥1 affected maxillary anterior teeth or ≥4 decayed, missing teeth due to caries, or restored tooth surfaces. The number of decayed (cavitated), missing teeth due to caries, or restored (filled) primary teeth (dmft) was also calculated.	Allocation status, taking into account maternal age, level of education, parity, prepregnancy body mass index, smoking status, and social class. Adjustments were also made for child sex, age at dental assessment, length-for-age Z-score (6 months), exclusive breastfeeding duration, and nursing bottle use (6 months).	3rd tertile 12-month sweet intake score x S-ECC RR1.55 (1.17. 2.23)95%CI	This study demonstrated a high prevalence of sugar-rich foods and beverages in the diet of many infants, with significant consequences for dental health as the number of sugar-rich items increased
			At the 6-month assessment, mothers were asked at what age (in months) their child was first introduced to 31 specific items, each of which was further categorized as introduced before 6 months (yes/no) and classified into two groups by cariogenic potential			3rd tertile 12-month sweet intake score x dmft RR1.78 (1.20, 2.90) 95%CI	
Chankanka et al., 2012, USA^23^	377 (Iowa Fluoride Study cohort)	36, 48, and 60 months	Dietary recall for two weekdays and one weekend day, along with the time of consumption at 36, 48 and 60 months	d1-d3 system modified based on the criteria proposed by Pitts for the age of 5 years	Average daily toothbrushing frequency, composite water fluoride levels and SES	Regular soda intake during snacks (occasions/week): d1d2+f versus caries-free: OR1.137 (0.889, 1.455) 95% CI Added sugar intake during snacks (occasions/week) d1d2+f versus caries-free: OR 1.623 (1.181, 2.230)95% CI	Higher frequencies of regular soda consumption and added sugar intake during snacks were significantly associated with classification into the cavitated caries (d2+f and/or d1d2+f ) groups.
Echeverria et al., 2022, Brazil^5^	2,806 (2015 Pelotas Birth Cohort)	3, 12, 24, and 48 months	The exposure variable of interest was the pattern of sugar consumption. The pattern of sugar consumption was obtained from the consumption of sugar at the 3-, 12-, 24-, and 48-month follow-ups.	ECC, the study outcome, was assessed on clinical examination using ICDAS. ECC was analyzed as two dichotomous outcomes: dental caries experience and cavitated dental caries.	Family income, maternal education, maternal age, and wheter the mother had received any oral health instruction from a health professional	Growing sugar consumption pattern x cavitated dental caries PR1.60 (1.33–1.92) 95%CI Growing sugar consumption pattern x dental caries experience PR 1.50 (1.29–1.74) 95%CI	An association was found between the pattern of sugar consumption and dental caries at 48 months. Children with increasing and persistently high sugar consumption have the highest prevalence of caries
Echeverria et al., 2023, Brazil^6^	3,654 (2015 Pelotas Birth Cohort)	3, 12, 24, and 48 months	The age at which sugar was first introduced into the child›s diet was considered the main exposure in the study.	Two outcomes were considered: 1) caries experience, including white spot lesions and cavitated lesions; and 2) cavitated lesions, assessed by the simplified ICDAS	family income at the time of birth, maternal education, and maternal age at delivery	Age at introduction of sugar <12 x dental caries experience: PR1.48 (1.13-1.95) 95%CI (0.98-1.93) 95%CI	An association was found between the age at which sugar was introduced and dental caries experience at 48 months.Caries experience was 48% higher in the group with sugar introduction before 12 months of age, compared to the group in which sugar was introduced after 24 months of age
			Based on questionnaire data, the timing of introduction of sugary foods and beverages in the child›s diet was categorized as before the age of 12 months, from 12 to 24 months, or after 24 months.	Age at introduction of sugar <12 x cavitated caries PR 1.38			
Feldens et al., 2010, Brazil^9^	340 children (prospective birth cohort in São Leopoldo – Brazil)	6, 12 months and 4 years of age	Monthly interviews investigated breastfeeding and bottle feeding during day and night, frequency and composition of complementary foods, as well as use of sugar, honey, sweetened beverages, biscuits, chocolate, and salty snacks. At 12 months, a 24-hour dietary recall was used by fieldworkers for obtaining data on the number of meals and snacks, frequency of breast- feeding, cow’s milk volume, use of bottles for liquids other than milk (generally fruit juices, beverages, or teas) and nighttime bottle use. At 12 months, the mothers were also asked about the intake of foods with high density of sugar in the past month. High density of sugar was regarded as 150% of simple carbo-hydrates in 100 g of food (e.g. candies, soft drink, sugar ,and honey)	S-ECC. The outcome in this study was S-ECC according to NIH case definition [Drury et al., 1999]: >= cavitated, missing or filled smooth surfaces in primary maxillary anterior teeth, or decayed (d1+), missing or filled surface (dmfs) values >=5.	child’s age and sex, maternal schooling, income per capita, toothbrushing with fluoride paste, and number of teeth at 12 months	RR 1.43; 1.08–1.89 95% CI	The results confirmed the hypothesis that some early-life feeding practices influence the severity of dental caries in subsequent years. Prevalence of S-ECC at 4 years of age is 43% higher in the group with consumption of high-density sugar
Grindfjord et al., 1996 , Sweden^25^	692 (living in eight suburbs of Stockholm)	2.5 and 3.5 years	Consumption of sugary beverages (<2/day, >2/day, 1/years, 2.5 years). Consumption of candies (<1/week, >1/week, 1/years, >1/years, 2.5 years)	Initial caries on smooth surfaces was recorded when the surface showed a loss of translucency and slight roughness on probing. Manifest caries was defined as the minimal level that could be verified as a cavity, detectable by probing, and as fissures, by a catch of the probe under slight pressure	Social and immigrant background, microbial profile, oral hygiene factors, and fluoride exposure	consumption of candy x Caries: OR 2.28 (1.28-4.04)95%CI	The incidence of caries was associated with the consumption of candy
Grytten et al., 1988, Norway^18^	231	6, 18, and 36 months	«Use of sweetened comforters» and «use of softdrinks at night-time «at the age of 6 months, frequencies of «soft drinks consumption» and «candy consumption» at the age of 18 months and «request for candy» at the age of 36 months. These variables were classified into an additive index, which was then divided into three categories on an ordinal scale: «Infrequent sugar consumption», «frequent sugar consumption», and «very frequent sugar consumption».	dmfs: divided into two categories: “0 dmfs” and “1 to 9 dmfs”	Use of of fluoride tablets and toothbrushing	Not reported	The frequency of sugar consumption was the only behavioral variable tested that showed a statistically significant association with caries experience at 36 months.
Ha et al., 2023, Singapore^24^	879	1, 2, and 5 years	At the age of one year, a 24-hour recall and 2-day diet diary was used to collect data on all foods and drinks consumed by the child. At the ages of two and five years, age-specific 98- and 99-item food frequency. questionnaires (FFQ) were used to collect detailed data on foods and drinks known to contain free sugars. Child daily free sugar intake (FSI) quantity was determined in grams for each of the three waves. The collected data on FSI at the three time points were then used in group-based trajectory modelling (GBTM) to characterize FSI trajectories.	Dental caries prevalence was measured as the percentage of children with at least one decayed, missing or filled tooth surface (dmfs), while cumulative dental caries experience was summarized as the dmfs score.	birth weight (<2,500; 2,500+ grams) and breastfeeding duration (<17; 17-25; 26-51; ≥52 weeks). mother’s age at birth (years); mother’s level of education (high school, vocational training, tertiary); area-based deprivation quintile (generated from the index of relative socioeconomic Advantage and disadvantage for areas, with the 1st quintile indicating the poorest area and the 5th quintile corresponding to the richest area, ; household annual income (≤AUD$40,000, >40, 000-80,000, >80,000-120,000, >120,000); mother’s country of birth (Australia/New Zealand and UK; India; Asia, except for India; others); household type (single-, two-parent household); child’s sex (female, male); and child’s age (in months).	RR 2.1 (1.3-3.3)	Evidence from this prospective birth cohort shows that a persistently high FSI trajectory from age 1 to 5 years was positively associated with dental caries at the age of 5 years.
Hu et al., 2019, Singpore^19^	363	6, 9, and 12 months (sugar) 2 and 3 years (ECC)	dietary recall - Each food item from the records was then assigned to one of the 72 subfoods from the 18 groups, based on food type or similarities in nutrient content, conceptually similar to previous studies Among the food groups, the confectionery and sugary drinks groups were selected for analysis. The confectionery food group consists of foods such as chocolates, candies, ice cream, puddings, and jellies, while the SSB consists of fruit drinks, carbonated soft drinks, sweetened milk, traditional drinks, and other sweetened beverages such as honey mixed with water.	ICDAS criteria at 2 and 3 years of age	Sociodemographic characteristics, oral hygiene habits, and perinatal and postnatal characteristics.	Confectionery consumption (Frequency) x ECC: OR1.05 (0.25 to 4.43 95% CI) Sugar-sweetened beverages (Frequency) x ECC: OR0.67 (0.42 to 1.09 95% CI) Confectionary (Amount in grams) x ECC: OR1.03 (0.93 to 1.15 95%IC) Sugar-sweetened Beverages (amount in grams) x ECC: OR 1.00 (0.99 to 1.00 95% CI)	When ECC status was analyzed at the age of 3 years, no significant association with frequency and amount of SSB with the presence or absence of ECC was observed. The trend was similar in relation to confectionery consumption. However, following the WHO-recommended weaning dietary pattern at 6 months and an increase in the dietary pattern score between 6 and 12 months were protective against the development of ECC.
Manohar et al., 2021, Australia^20^	738	4, 8, 12, 24, and 36 months	Children’s dietary habits, in terms of consumption of 32 individual food and drink items in the preceding seven days, were recorded using a short food frequency questionnaire (FFQ). For dietary trajectory analyses, the 32 listed food and drink items were broadly categorized into ‘core’ and ‘discretionary’ food groups based on the 2013 Australian Dietary Guidelines. Additionally, the discretionary food group was further categorized into the sugary food group (n = 18 items). The frequency (continuous data) of each item in the five individual core food subgroups were summed to obtain the ‘total of the core food group intake’, and the frequency of each item in the two discretionary food groups were summed to obtain the ‘total of the discretionary food group intake’. This same method was used for sugar-containing items to obtain the ‘total of the sugary food group intake’.	dmfs: ECC was characterized as the ‘presence of one or more decayed (non-cavitated or cavitated lesions), missing (due to decay), or filled tooth surfaces in any primary (baby) teeth in children less than 6 years of age	Participant characteristics—dietary trajectories, demographic, socioeconomic, and behavioral variables, overweight, and obesity.	Dietary trajectories Sugary foods (high and late declining trajectory) x ECC: IRR0.90 (0.47 -1.70 95% CI)	No statistically significant or clinically meaningful association was found between trajectories of sugary food intake and ECC after adjusting for covariates.
Peltzer et al., 2015, Thailand^26^	597	12 and 30 months	dietary recall - A sugary food consumption index was created, including five items: sweet jelly, sweet drink, sweet candy, sweet	dmfs: The outcome of this study was S-ECC, defined as ≥1 cavitated, missing or filled smooth surfaces in primary maxillary anterior teeth, or decayed, missing or filled surface (dmfs) values ≥4.	Sociocultural risk factors and environmental risk factors.	Not reported	Eating sweet foods more often was associated with severe caries in early childhood
Persson et al., 1985, Sweden^21^	312	12 and 36 months	Information on dietary habits at the age of 12 months was collected by trained dieticians through home-based interviews. The questionnaire recorded the frequency of intakes on an eight-category scale ranging from «never» to «4 times per day or more»	surfaces with manifest lesions were recorded as carious	Not reported	Not reported	At the age of 1 year, children with caries by the age of 3 years had generally consumed cakes, butter, bread, and sweet soups more frequently. Some «staple» foods (porridge and follow-up formula, and meat) were consumed more frequently in the non-caries group
Thitasomakul et al., 2009, Thailand^22^	495	9, 12, and 18 months	An interview was conducted when the infant was 3 months old to collect data on the type of milk-feeding, infant supplementary food such as cooked rice and commercial cereal, child’s age when the mother introduced sugary foods, and the age at which the child began eating snacks, vegetables, and fish. These data were re-collected at the ages of 9 and 12 months.	dental status of each examined surface as: (1) unerupted tooth, (2) normal enamel surface, (3) initial caries or caries limited to enamel, (4) caries extending into dentin, and (5) caries with pulpal involvement.	income, level of education, supplementary calcium use, and milk intake during pregnancy	Child started eating sugary snacks at 9 months x ECC: IDR1.2 Child started consuming soft drinks at 9 months x ECC: IDR1.3	A significantly higher crude caries increment among children who were breast-fed, had sugary snacks, and had local traditional desserts at 9 months, among those who started eating vegetables after 6 months of age
Watanabe et al., 2014, Japan^27^	31,202	1.5 and 3 years	The daily frequency of sugary snack intake was classified as none, once a day, twice a day, and three times or more a day.	The presence or absence of dental caries, including initial carious lesions, was recorded for all erupted tooth surfaces. Initial caries was defined as a demineralized surface with a chalky appearance, but without macroscopic loss of tooth substance, whereas manifest caries was defined as the minimal level that could be verified as a cavity on probing	Nationality, sex, order of birth, cariostat score	Daily sugar-sweetened beverage consumption OR1.56 (1.46, 1.65)95%CI	Children who consumed sugary snacks 3 times a day increased their risk of developing dental caries by 3.9-fold (OR: 3.90; 95% CI: 2.79, 5.45) (4.6-fold in boys; 3.2-fold in girls compared with children who did not consume sugary snacks at the age of 1.5 years. The OR of caries for consumption of sugary snacks (OR: 2.0, 3.90) was higher than that of sugar-sweetened beverages (1.56), suggesting that sugary snacks may be a more important determinant of early childhood caries.
Wendt et al., 1995, Sweden^31^	593	1, 2, and 3 years	Parents were asked about the irchildren›s dietary habits during the past year, namely, i)breast-feeding habits, ii) bottle-feeding habits, iii) nocturnal meals, iv) regular meals during the daytime, v) eating problems, vi) what kind of food the child preferred to eat, vii)number of daily intakes of caries-risk products, such as a feeding bottle with sugar-containing liquid, soft drinks, fruit soup, sweets, ice cream, or biscuits. The intake frequency of each caries-risk product was assigned points: 7 points indicated once a day, 1 point once a week, and so forth. The sum of the points for each child was then divided by 7 to calculate the average number of intakes of caries-risk products per day.	The presence of caries, including initial carious lesions, was clinically diagnosed on all tooth surfaces by visual examination and probing. Radiographic examinations were performed on 3-year-olds when proximal contacts precluded clinical examination.	Not reported	Not reported	Children with caries at 2 and 3 years of age and immigrant children had, when they were 1 year old, consumed caries-risk products and been given nocturnal meals and sweetened liquid in a feeding bottle more often than caries-free 2- and 3-year-olds and non-immigrant children.

**Table 3:** Quality assessment of the selected studies using the JBI criteria.

Authors	Were the two groups similar and recruited from the same population?	Were the exposures measured similarly to assign people to both exposed and unexposed groups?	Was the exposure measured in a valid and reliable way?	Were confounding factors identified?	Were strategies to deal with confounding factors stated?	Were the groups/participants free of the outcome at the start of the study (or at the moment of exposure)?	Were the outcomes measured in a valid and reliable way?	Was the follow-up time reported and sufficient to be long enough for outcomes to occur?	Was follow-up complete, and if not, were the reasons to loss to follow-up described and explored?	Were strategies to address incomplete follow-up utilized?	Was appropriate statistical analysis used?	Risk of bias
Amezdroz et al., 2019^16^	Yes	Yes	Yes	No	No	Unclear	Yes	Yes	Yes	No	Yes	Low
Bernabe et al., 2020^7^	Yes	Yes	Yes	Yes	Yes	Unclear	Yes	Yes	No	No	Yes	Low
Chaffee et al., 2015^8^	Yes	Yes	Yes	Yes	Yes	Yes	Yes	Yes	Yes	No	Yes	Low
Chankanka et al., 2015^23^	Yes	Yes	Yes	Yes	Yes	Yes	Yes	Yes	Yes	No	Yes	Low
Echeverria et al., 2022^5^	Yes	Yes	Yes	Yes	Yes	Yes	Yes	Yes	Yes	No	Yes	Low
Echeverria et al., 2023^6^	Yes	Yes	Yes	Yes	Yes	Yes	Yes	Yes	Yes	No	Yes	Low
Feldens et al., 2010^9^	Yes	Yes	Yes	Yes	Yes	Yes	Yes	Yes	Yes	No	Yes	Low
Grindfjord et al., 1996^25^	Yes	Yes	Yes	Yes	No	Yes	Yes	Yes	Yes	No	Yes	Low
Grytten et al., 1988^18^	Yes	Yes	Yes	No	No	Unclear	Yes	Yes	Yes	No	Yes	Low
Ha et al., 2023^24^	Yes	Yes	Yes	Yes	Yes	Yes	Yes	Yes	Yes	No	Yes	Low
Hu et al., 2019^19^	Yes	Yes	Yes	Yes	Yes	Unclear	Yes	Yes	Yes	No	Yes	Low
Manohar et al., 2021^20^	Yes	Yes	Yes	Yes	Yes	Unclear	Yes	Yes	Yes	No	Yes	Low
Peltzer et al., 2015^26^	Yes	Yes	Yes	Yes	Yes	Unclear	Yes	Yes	Yes	No	Yes	Low
Persson et al., 1985^21^	Yes	Yes	Yes	No	No	Unclear	Yes	Yes	Yes	No	Yes	Low
Thitasomakul et al., 2009^22^	Yes	Yes	Yes	Yes	Yes	Yes	Yes	Yes	Yes	No	Yes	Low
Watanabe et al., 2014^27^	Yes	Yes	Yes	Yes	Yes	Yes	Yes	Yes	Yes	No	Yes	Low
Wendt et al., 1995^31^	Yes	Yes	Yes	No	No	Unclear	Yes	Yes	Yes	No	Yes	Low

A meta-analysis involving all included studies was not performed due to inconsistencies in the reporting of results. Nine studies, however, provided adjusted effect measures (RR, PR, or OR) and reported the prevalence of caries in the group not exposed to sugar. This allowed the pooled data in the meta-analysis to be expressed as OR.

**Figure 2:**
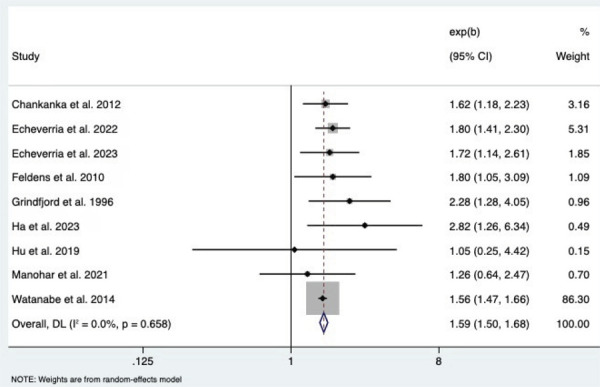
Meta-analysis evaluation and forest plot showing the combined odds ratio of all included studies.

The pooled (meta-analyzed) effect estimated in this study yielded an OR of 1.59 (95%CI:1.50–1.68). This finding indicates that children who consumed sugar in early childhood were 59% more likely to develop caries compared to those who did not consume it, with a statistically significant overall effect. Furthermore, the Cochran’s Q value of 5.91, p = 0.658, indicates that the heterogeneity test was not significant, i.e., the effects do not differ more than expected by chance. Figure 2 shows the pooled effect of sugar consumption in longitudinal studies and the occurrence of dental caries.

## Discussion

The association between sugar consumption and ECC is consistent across the cohort studies included in this review, with most reporting that higher sugar consumption is associated with higher prevalence of dental caries. The meta-analysis yielded a pooled effect estimate of OR 1.59 (95%CI: 1.50–1.68), demonstrating that children who consumed sugar at an early age were almost 60% more likely to have dental caries than those who did not.

A recent large birth cohort study found that higher sugar consumption in the first 48 months of life was strongly associated with a higher prevalence of dental caries at the age 4 years.^5^

Sugar exposure before the age of 1 year was reported in 11 studies.^5,6,8,16-22^ Previous studies have reported a high percentage of children consuming sugary foods and beverages by the end of their first year of life.^28^ The early exposure of a child to sucrose influences their dietary preferences, stimulating their preference for foods and drinks containing added sugars over healthier foods,^29^ which may, in turn, contribute to caries experience in the future. Moreover, early exposure to sugary foods could influence similar dietary patterns in the subsequent stages of life,^5,8,9^ increasing the risk for several chronic diseases over the couse of life, including ECC.^11^ Hence, the IAPD recommends that sugar intake should be limited among children under 2 years of age.^9,30^ A birth cohort study also showed that the early introduction of sugar is associated with a higher incidence of dental caries.^6^

The high frequency of sugars and sugar sweetened beverages was associated with ECC in eight studies.^16,18,23-27,31^ The feeding frequency could play a role in caries development, independently of the amount of potentially cariogenic foods consumed.^8,30,32^ These findings underscore the importance of identifying risk factors related to eating behaviors in early childhood to prevent caries in children through changes in the diet.

As noted in this review, cohort studies apply various methods for assessing exposure to sugary foods and beverages ,which should be considered when comparing their results. Different instruments were used in the original studies included in this review, but the most frequent were those that measure the daily frequency of sugary foods or beverages,^5,6,19,23,27^ followed by the assessment of sugar intake using food diaries with a 24-hour recall,^19,26^ and weekly frequency of sugar consumption^16,25^ There is a vast literature on ECC, and many studies assess other major exposures. Those studies that use sugar consumption only as a covariate, despite their relevance to the literature, were not included in this review because most lacked detailed information on how sugar consumption was measured.

The current review has several strengths that should be considered, such as the use of multiple databases and the inclusion of only longitudinal studies, in which the temporal relationship between exposure and outcome could be observed. In addition, the review protocol was previously registered, the review process and data extraction were performed independently by two reviewers, and the study was reported following the most recent guidelines. Also, a meta-analysis was carried out with nine studies, and the effect measured confirmed the findings of the qualitative analysis. This review has certain limitations, including the variability in dietary data collection methods, which limited the feasibiity of conducting a meta-analysis,and the lack of studies from low-income countries.

There is a clear need to enhance the quality of dietary data collection. The use of different methods to evaluate sugar intake hinders the comparison between different studies and it is a strong limitation of the studies evaluating diet and health outcomes. Future studies should focus on the development of more accurate methods to measure sugar intake, thus representing a strong contribution to the field.

This review highlights the importance of developing strategies and policy interventions at the population level to reduce sugar consumption during childhood. Therefore, efforts are needed to limit the amount and frequency of sugar consumption, such as promoting sugar-free environments in schools, pre-schools, and workplaces; regulating added sugar content in foods and beverages; restricting advertisement of sugary products; implementing higher taxes on sugary foods and beverages; reformulating the sugar content of foods and beverages; and lowering sugar concentration.^9^ Furthermore, dentists, when providing personalized care to prevent ECC, may contribute to decreasing the consumption of sugar by implementing educational interventions targeted at individuals and their communities,^9^ as well as providing guidance to parents and families about the cariogenic potential of sugar consumption and frequent feeding.

All studies included in this review demonstrated that sugar was introduced into the infant’s diet too early (before the age of 24 months). This finding goes against dietary guidelines for healthy eating, possibly revealing parents’ lack of knowledge about proper nutrition for their children, socioeconomic problems that restrict access to healthy foods, and cultural influences on family eating practices. Therefore, it is necessary that health professionals address breastfeeding and healthy eating following WHO and IAPD guidelines.^10-11^ Educating parents and caregivers about the importance of avoiding sugary foods early in life is crucial for preventing many chronic diseases associated with high sugar consumption, including but not restricted to ECC.

## Conclusion

This systematic review and meta-analysis confirmed the association between sugar consumption and ECC using data from good-quality longitudinal cohort studies.

## Data Availability

The authors declare that all data generated or analyzed during this study are included in this published article.
